# Optimally Tuned Multiconfigurational Short-Range DFT for Linear Response Properties

**DOI:** 10.1021/acs.jpca.6c01551

**Published:** 2026-05-08

**Authors:** Michal Hapka, Katarzyna Pernal, Ewa Pastorczak

**Affiliations:** † Faculty of Chemistry, 49605 University of Warsaw, ul. L. Pasteura 1, Warsaw 02-093, Poland; ‡ Institute of Physics, 49584 Lodz University of Technology, ul. Wolczanska 217/221, Lodz 93-005, Poland

## Abstract

Multiconfigurational short-range density functional theory (MC-srDFT) rigorously combines ground-state wave function theory with DFT. Unlike single-reference range-separated hybrid functionals, MC-srDFT has lacked theoretically grounded protocols for choosing the system-specific range-separation parameter. To address this problem, we introduce an optimal-tuning scheme based on enforcing the correct exponential decay of the electron density. We show that the range-separation parameter can be determined from the ionization potential given by the smallest-magnitude eigenvalue of the Extended Koopmans’ Theorem matrix constructed for the model Hamiltonian. We validate this approach for static and dynamic dipole polarizabilities of ground-state molecular systems using MC-srDFT within both full linear response and its extended random phase approximation (ERPA) variant. Optimal tuning substantially improves polarizabilities relative to the commonly used universal μ = 0.4 bohr^–1^ parameter.

## Graphical abstract

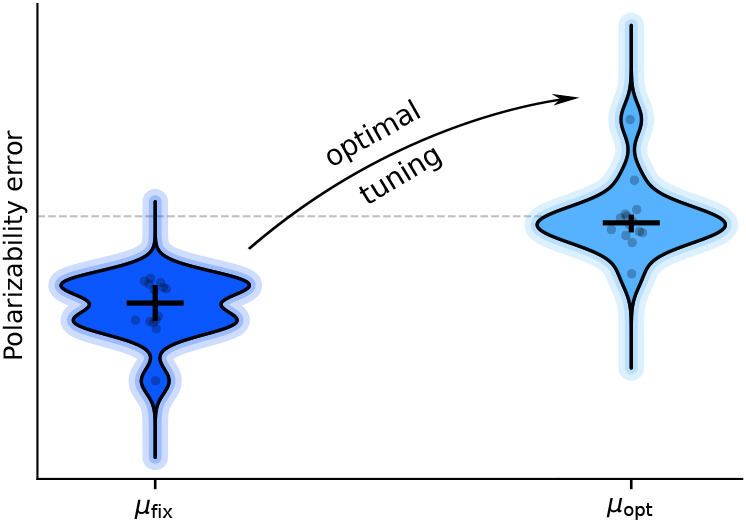

## 1 Introduction

Wave function theory (WFT) and DFT can be rigorously combined in a way that benefits both approaches, even when approximate treatments are employed. A prominent example is provided by range-separated methods, in which the electron–electron interaction is decomposed into short- and long-range components treated by DFT and WFT, respectively.^1,2^ This leads to range-separated multiconfigurational DFT (MC-srDFT), originally proposed by Savin et al.^3–5^ to address the inability of standard DFT approximations to describe ground states of near-degenerate systems. Soon after its introduction, MC-srDFT was recognized as an efficient route for incorporating dynamic correlation into wave function methods.^5^ By assigning short-range correlation to density functionals, the required wave function expansions can be kept compact, thereby reducing sensitivity to the choice of active space in complete active space (CAS) methods and to excitation-level truncation in configuration interaction^6,7^ and coupled-cluster^8,9^ theories.

To date, most MC-srDFT applications have focused on ground- ^10–12^ and excited-state energies,^13–19^ demonstrating promising accuracy. Nevertheless, a central challenge of the approach—the dependence on the range-separation (RS) parameter μ that governs the partitioning of the Coulomb operator—has not been fully resolved. Fromager et al.^20^ advocated the use of a universal value of μ, chosen to preserve single-reference character in systems dominated by dynamic correlation while recovering essential static correlation effects in the multireference regime. Based on studies of ground states of small atoms and molecules,^20,21^ they recommended μ = 0.4 bohr^–1^. On the one hand, the use of a universal, global μ guarantees size consistency and avoids system-specific parameter tuning. On the other hand, a systematic protocol for such tuning has not been developed within MC-srDFT. This is in stark contrast to single-reference range-separated DFT, where optimal tuning strategies are well established. These include either ionization potential (IP) tuning,^22–24^ which enforces the Koopmans condition,^25,26^ $\varepsilon_{\mathrm{HOMO}}^{\mathrm{KS}}=-\mathrm{IP}$ , which is exact in Kohn–Sham (KS) DFT, or global density-dependent tuning^27,28^ in which the range-separation parameter becomes a functional of the density, μ ≡ μ­[ρ].

Relatively few works so far have employed time-dependent linear-response extensions of MC-srDFT^13,29^ to the calculation of response properties. Hedegård assessed the accuracy of CAS-srDFT oscillator strengths^17^ for the Thiel set^30^ obtaining satisfactory agreement with CC2 results. Jensen and coworkers^31,32^ showed that both CAS-srDFT and GVB-srDFT (GVB denoting generalized valence bond wave function) give reliable predictions of indirect spin–spin coupling constants. Their work is a good example of MC-srDFT sensitivity to the RS parameter value: for transition-metal complexes, satisfactory results were obtained by setting μ to 1.0 bohr^–1^ instead of the standard 0.4 bohr^–1^ choice.

In this work, we introduce a rigorous and physically motivated procedure for determining the range-separation parameter, which, to the best of our knowledge, has not been previously formulated within the MC-srDFT framework. The proposed tuning criterion is based on an exact extension of the Koopmans condition to MC-srDFT and on the requirement that optimally tuned MC-srDFT, employing approximate short-range exchange–correlation functionals and approximate wave functions, reproduces the correct long-range asymptotic decay of the electron density.

We assess the performance of the optimally tuned MC-srDFT approach by computing static and dynamic molecular polarizabilities. Dipole polarizabilities are known to be sensitive to both the quality of the electronic structure description and the employed basis set.^33,34^ In particular, experience with range-separated hybrid (RSH) DFT functionals demonstrates a pronounced dependence of polarizabilities on the value of the RS parameter,^35–38^ which becomes even more significant for hyperpolarizabilities.^39–44^ It is worth noting that asymptotically corrected exchange-correlation potentials^45–47^ offer an alternative way of improving DFT response properties by enforcing the correct density decay. Such potentials have found particularly extensive application within symmetry-adapted perturbation theory.^48,49^

In addition to dipole polarizabilities computed from the full linear-response MC-srDFT formalism,^13^ we examine the performance of the extended random phase approximation (ERPA)^29,50,51^ applied to MC-srDFT wave functions. In contrast to the full linear response, ERPA neglects contributions from the response of the wave function expansion coefficients, accounting solely for orbital relaxation. This simplification leads to a more favorable scaling with respect to the size of the active space.

The paper is organized as follows. In Section 2, we briefly recapitulate the MC-srDFT formalism and introduce the proposed optimal tuning protocol. We then present ERPA-based linear response equations for the calculation of dipole polarizabilities in the MC-srDFT framework. In Section 3, the performance of the employed linear-response approaches is benchmarked against CC3 dipole polarizability reference data for the test set of Jørgensen et al.^52^ Finally, Section 4 summarizes the main findings and outlines perspectives for further methodological development and applications.

## 2 Theory

### 2.1 Range-Separated Multicofigurational DFT (MC-srDFT)

The multiconfigurational density functional theory, denoted here as MC-srDFT, proposed by Savin et al.^3,5,53,54^ cf. also refs 1 and 2, is based on the separation of the electron–electron Coulomb interaction operator, $r_{12}^{-1}$ , into short-range (SR) and long-range (LR) components, ${\mathrm{\upsilon ̂}}_{ee}^{\mathrm{SR}}\left(r_{12}\right)$ and ${\mathrm{\upsilon ̂}}_{ee}^{\mathrm{LR}}\left(r_{12}\right)$ , respectively. The essential conditions for the long-range part are that it is finite at the electron–electron coalescence, *r*_12_ → 0, and reduces to the Coulomb interaction, 1/*r*_12_, in the large-separation limit. The exact ground-state range-separated density functional takes the form

1 $$E^{\mathrm{MC‐srDFT}}\left[\Psi \right]=\left\langle \Psi \right|\mathrm{T̂}+{\mathrm{V̂}}_{ne}+{\mathrm{V̂}}_{ee}^{LR}\left|\Psi \right\rangle +E^{SR}\left[\rho_{\Psi }\right]$$

where T̂ and ${\mathrm{V̂}}_{\mathrm{ne}}$ are, respectively, the kinetic energy and electron-nuclei interaction operators, ρ_Ψ_ stands for electronic density corresponding to a wave function Ψ. eq 1, in principle, rigorously defines the short-range functional *E* ^SR^[ρ], but the exact form of the latter is unknown. For practical approximations, *E* ^SR^[ρ] is typically split into a Hartree functional, $E_{H}^{\mathrm{SR}}\left[\rho \right]=\frac{1}{2}\int \int \rho \left(r_{1}\right){\mathrm{\upsilon ̂}}_{ee}^{\mathrm{SR}}\left(r_{12}\right)\rho \left(r_{2}\right)dr_{1}dr_{2}$ , and the exchange-correlation functional $E_{xc}^{\mathrm{SR}}\left[\rho \right]$ .^54,55^

Ground state energy follows from minimization of the MC-srDFT functional, which is equivalent to solving an eigenproblem for the Hamiltonian *Ĥ* ^LR^

2 $${Ĥ}^{LR}\Psi^{LR}=E^{LR}\Psi^{LR}$$

where *Ĥ* ^LR^ includes the long-range electron–electron interaction operator

3 $${Ĥ}^{LR}=\mathrm{T̂}+{\mathrm{V̂}}_{ne}+{\mathrm{V̂}}_{ee}^{LR}+{\mathrm{V̂}}^{SR}\left[\rho_{\Psi^{LR}}\right]$$

and the short-range local potential ${\mathrm{V̂}}^{\mathrm{SR}}$ , which follows as a functional derivative of *E* ^SR^[ρ]. The wave function Ψ^LR^ differs from the exact ground state correlated wave function (unlike the latter, for example, the former is free of the electron coalescence cusp). Contrary to this, the electron density ρ_Ψ^LR^_ calculated from the wave function Ψ^LR^ coincides with the exact ground state density, ρ_0_, as in the conventional DFT

4 $$\rho_{0}(r)=\rho_{\Psi^{LR}}(r)$$

on the condition that the SR functional is exact.

The most successful approximations to the SR exchange-correlation functionals have been developed for the error-function representation of the LR electron interaction^7,9,56,57^

5 $${\mathrm{\upsilon ̂}}_{ee}^{LR}\left(r_{12}\right)=\frac{\mathrm{erf}\left(\mu r_{12}\right)}{r_{12}}$$

which involves a range-separation parameter, μ. By varying μ from 0 to ∞, one smoothly switches between the KS-DFT and full-range-interaction-FCI limiting cases.

In contrast to MC-srDFT with the exact SR functional, whose energy is independent of the range-separation parameter, the use of approximate functionals makes MC-srDFT predictions dependent on this choice. In principle, more accurate functional approximations should be less sensitive to the choice of μ. Early investigations of the energies of atoms, small molecules, and dissociation energy curves suggested the value of the “universal” μ equal to 0.4 au.^20^ In practice, choosing the optimal value of μ has remained an open problem, especially for excited-state energies^12,19^ and for the response properties^31,32^ such as molecular polarizability.

### 2.2 Optimal Range-Separation Parameter for MC-srDFT Based on an Exact Condition

Recall that if the exact SR functional were available, the wave function Ψ^LR^ resulting from MC-srDFT, see eq 2, would still be different from the FCI wave function corresponding to the full-range electron interaction. However, the ground state electron density would be exact, see eq 4. It would therefore decay exponentially with the distance *r* ≡ |**r**| from the system as

6 $$\rho_{\Psi^{LR}}\left(r\to \infty \right)∼\exp\left[-2\sqrt{2{\mathrm{IP}}_{1}}r\right]$$

Importantly, the IP_1_ denotes the first ionization potential of the real system, i.e., the one described by the Hamiltonian with full-range electron interaction. It is therefore different from the ionization potential of a reference system described with a model Hamiltonian, *Ĥ* ^LR^.

Using approximate SR functionals in MC-srDFT violates the equality assumed in eq 4 and, in consequence, yields erroneous asymptotic decay of the density. By using arguments of the seminal paper by Morrell, Parr, and Levy (MPL),^58^ one can show how to tune the RS parameter μ so that the approximate electron density decays exponentially with the exact ionization potential of a given system.

We begin by generalizing the derivations presented by MPL to model Hamiltonians. Assume a model (*M*) Hamiltonian involving a modified electron interaction operator $\upsilon_{ee}^{M}$ , one-electron kinetic energy operator *t̂*(**r** *_i_*), and a model external potential ${\mathrm{\upsilon ̂}}_{ext}^{M}$

7 $${Ĥ}^{M}=\overset{N}{\underset{i=1}{\sum }}\left(\mathrm{t̂}\left(r_{i}\right)+{\mathrm{\upsilon ̂}}_{ext}^{M}\left(r_{i}\right)\right)+\frac{1}{2}\overset{N}{\underset{i\neq j}{\sum }}\upsilon_{ee}^{M}\left(\left|r_{i}-r_{j}\right|\right)$$

giving rise to a model electron density

8 $$\rho^{M}\left(x\right)=N\int |\Psi^{M}\left(x,x_{2},...,x_{N}\right){|}^{2}dx_{2}...dx_{N}$$

where Ψ*^M^* is a wave function corresponding to *Ĥ* ^*M*^, and *x* denotes a combined spin-spatial coordinate.

Provided that (*a*) ${\mathrm{\upsilon ̂}}_{ext}^{M}\left(r\right)$ is a local potential vanishing at *r* → ∞, and (*b*) $\upsilon_{ee}^{M}\left(r\right)$ decays as a Coulomb potential in the long-range

9 $$\upsilon_{ee}^{M}\left(r\to \infty \right)∼\frac{1}{r}$$

one can repeat the arguments of MPL [see eqs (43)−(58) in ref 58] and arrive at the asymptotic decay of a model electron density reading

10 $$\rho^{M}\left(r\to \infty \right)∼\exp\left[-2\sqrt{-2\lambda_{\max}^{M}}r\right]$$

On the same basis, the long-range behavior of the natural orbitals (NOs) $\left\{\varphi_{p}^{M}\left(x\right)\right\}$ , diagonalizing a model one-electron reduced density matrix (1-RDM), γ^*M*^

11 $$\gamma^{M}\left(x,x^{\prime }\right)=\underset{p}{\sum }n_{p}^{M}\varphi_{p}^{M}{\left(x^{\prime }\right)}^{*}\varphi_{p}^{M}\left(x\right)$$

is obtained, reading

12 $$\forall_{p},\varphi_{p}^{M}\left(r\to \infty \right)∼\exp\left[-\sqrt{-2\lambda_{\max}^{M}}r\right]$$

The number $\lambda_{\max}^{M}$ is the largest (least negative) eigenvalue of the matrix **Λ** ^*M*^ defined for a given model Hamiltonian. In the natural-orbitals representation, it takes the form

13 $$\sqrt{n_{p}^{M}n_{q}^{M}}\Lambda_{pq}^{M}=n_{q}^{M}\left\langle \varphi_{p}^{M}\right|\mathrm{t̂}+{\mathrm{\upsilon ̂}}_{ext}^{M}\left|\varphi_{q}^{M}\right\rangle +2\underset{rst}{\sum }\Gamma_{stqr}^{M}\left\langle \varphi_{p}^{M}\varphi_{r}^{M}\right|\upsilon_{ee}^{M}\left|\varphi_{s}^{M}\varphi_{t}^{M}\right\rangle$$

14 $$\lambda_{\max}^{M}=\max\left\{\lambda_{p}\right\}$$

where λ_*p*_ are eigenvalues of **Λ** ^*M*^, $\left\{n_{p}^{M}\right\}$ denotes natural occupation numbers, and Γ^*M*^ is the two-particle reduced density matrix, all corresponding to the model wave function Ψ*^M^*. In eq 13, we adopt physicist notation for two-electron integrals. The matrix **Λ** ^*M*^ has been known as the Extended Koopmans’ Theory (EKT) matrix.^59,60^ Thus, any model density corresponding to a Hamiltonian with modified electron interactions of the Coulomb tail decays exponentially, and the pace of this decay is governed by the first EKT eigenvalue which, in general, is specific to the assumed model.

Let us apply the general considerations for model Hamiltonians to MC-srDFT. By combining eqs 6 and 10, we obtain an exact and nontrivial result that the largest eigenvalue, $\lambda_{\max}^{M}$ , of the LR-EKT matrix, i.e., the matrix given by eq 13 computed with a model Hamiltonian matrix and reference RDMs corresponding to a model wave function Ψ^LR^, is equal to IP_1_ of a Coulomb system

15 $$-\lambda_{\max}^{LR}={\mathrm{IP}}_{1}$$

This property can be seen as a multiconfigurational generalization of the HOMO condition, i.e., equality of the negative HOMO orbital energy in the Kohn–Sham model and the exact ionization potential,^25,26^ $-\varepsilon_{\mathrm{HOMO}}^{\mathrm{KS}}={\mathrm{IP}}_{1}$ . If $\forall r_{12}\;{\mathrm{\upsilon ̂}}_{ee}^{\mathrm{LR}}\left(r_{12}\right)=0$ , then MC-srDFT turns to KS-DFT, eigenvalues of LR-EKT are just energies of the occupied KS orbitals, in particular $\lambda_{\max}^{\mathrm{LR}}=\varepsilon_{\mathrm{HOMO}}^{\mathrm{KS}}$ , and the condition in eq 15 is trivially satisfied. We have just shown, however, that it is satisfied for any form of ${\mathrm{\upsilon ̂}}_{ee}^{\mathrm{LR}}\left(r_{12}\right)$ .

Another feature of MC-srDFT, not fully recognized although straightforward to show from the discussion above, concerns the natural orbitals, $\left\{\varphi_{p}^{\mathrm{LR}}\right\}$ , that diagonalize the 1-RDM pertaining to Ψ^LR^. These orbitals are neither KS orbitals nor exact natural orbitals corresponding to the full-range electron interaction. Following MPL, one concludes that they decay asymptotically with $\lambda_{\max}^{\mathrm{LR}}$ . Taking eq 15 into account, this leads to the following asymptotic behavior

16 $$\varphi_{p}^{LR}\left(r\to \infty \right)∼\exp\left[-\sqrt{-2{\mathrm{IP}}_{1}}r\right]$$

Consequently, all MC-srDFT natural orbitals possess the same rate of decay as the exact ones.

The analysis above pertains to exact MC-srDFT. We now consider an MC-srDFT model employing an approximate SR functional with the erf-modified electron interaction, eq 5. Imposing the condition in eq 15, which is satisfied by exact MC-srDFT, uniquely fixes the range-separation parameter μ. The optimally tuned parameter μ_opt_ satisfies the equation

17 $$-\lambda_{\max}^{LR}\left(\mu_{opt}\right)={\mathrm{IP}}_{1}$$

Whether IP tuning is feasible depends on the particular form of *Ĥ^M^* (*μ*). If it leads to densities that differ significantly from the exact one, eq 17 may have no solution.

While the improved accuracy of computations employing the optimal value of μ instead of the conventional value of 0.4 bohr^−1^ is not guaranteed, we show in the next section that this is indeed the case for ground-state polarizability calculations. The improvement is likely related to the fact that the natural orbitals obtained from Ψ^LR^ with μ_opt_ show a proper LR decay, see eq 16, resembling the exact NOs’ LR behavior.

### 2.3 Approximate Dynamic Linear Response from MC-srDFT

The ground-state MC-srDFT theory can be extended to the time-dependent (TD) regime. The exact TD-MC-srDFT formalism was derived by Fromager and coauthors^13^ in the framework of the Floquet theory. Implemented in the adiabatic approximation for the short-range exchange-correlation kernel,^14^ it has given rise to time-dependent MC-srDFT linear response equations.

Another route to deriving an approximate linear-response TD-MC-srDFT function is to employ the equations-of-motion formalism of Rowe^61^ in the extended random phase approximation.^29,50^ Since our focus is on second-order response properties, we present below the relevant equations in the ERPA-MC-srDFT framework.

Consider a frequency-dependent linear response density function χ­(ω), defined as

18 $$\chi_{pq,rs}\left(\omega \right)=2\underset{I\neq 0}{\sum }\frac{\omega_{I}\left\langle \Psi_{0}\right|{â}_{q}^{†}{â}_{p}\left|\Psi_{I}\right\rangle \left\langle \Psi_{I}\right|{â}_{r}^{†}{â}_{s}\left|\Psi_{0}\right\rangle }{\omega^{2}-\omega_{I}^{2}}$$

It yields the response of the density matrix to a real one-electron perturbation *δv̂*, which in the frequency domain is given by

19 δγpqRe(ω)=∑rsχpq,rs(ω)δvrs(ω)

where *δγ* ^Re^(ω) is the Fourier transform of the real part of the time-dependent response density matrix *δγ*(*t*).

Combining the ERPA formalism for the LR Hamiltonian *Ĥ* ^LR^ presented in ref 19 with the ERPA density response equation derived in ref 62, we arrive at the following equation for the ERPA-MC-srDFT response function $\chi_{\mathrm{ERPA}}^{\mathrm{LR}}\left(\omega \right)$

20 $$\left[\omega^{2}-A_{+}^{LR}N^{-1}A_{-}^{LR}N^{-1}\right]\chi_{\mathrm{ERPA}}^{LR}\left(\omega \right)=A_{+}^{LR}$$

where, in the representation of the natural spinorbitals corresponding to Ψ^LR^,

21 $${[N]}_{pq,rs}=\delta_{pr}\delta_{qs}\left(n_{p}^{LR}-n_{q}^{LR}\right)$$

and

22 $$A_{+}^{LR}=A^{LR}+B^{LR}$$

23 $$A_{-}^{LR}=A^{LR}-B^{LR}+NKN$$

The matrices **A** ^LR^ and **B** ^LR^ are defined for the Hamiltonian *Ĥ* ^LR^ and the wave function Ψ^LR^ as

24 $$A_{pqrs}^{LR}=B_{pqsr}^{LR}=\left\langle \Psi^{LR}\right|\left[{â}_{p}^{†}{â}_{q},\left[{Ĥ}^{LR},{â}_{s}^{†}{â}_{r}\right]\right]\left|\Psi^{LR}\right\rangle$$

and are expressible in terms of 1- and 2-RDMs associated with Ψ^LR^, cf. ref 19. The matrix **K** represents a short-range kernel obtained from a derivative of the SR potential, cf. eq 3, with respect to the electron density

25 $$K_{pqrs}=\left\langle \varphi_{p}^{M}\varphi_{q}^{M}\left|\frac{\delta^{2}E^{SR}\left[\rho \right]}{\delta \rho \left(r_{1}\right)\delta \rho \left(r_{2}\right)}\right|\varphi_{r}^{M}\varphi_{s}^{M}\right\rangle$$

The ERPA-MC-srDFT linear response can be viewed as an approximation to the TD-MC-srDFT formalism of Fromager et al.^13^ TD-MC-srDFT reduces to the ERPA-MC-srDFT equations under the assumption that the time-dependent perturbation does not induce relaxation of the configuration-interaction (CI) coefficients, i.e., that only orbital rotations are allowed to respond. For short configuration-expansions of the wave function, one expects that both approximations would yield similar results, as confirmed in the next section.

While TD-MC-srDFT involves coupled orbital and configuration response equations whose dimensionality scales with the size of the CI expansion, ERPA restricts the problem to the orbital-rotation space. As a result, the dimension of the response matrix is dramatically reduced, and the computational cost becomes polynomial in system size rather than combinatorial in active-space size. The computational cost of a direct implementation of ERPA-MC-srDFT scales with the sixth power of the system size.

In practice, TD-MC-srDFT equations are solved using direct iterative techniques^63,64^ that avoid the explicit construction and storage of the **A**_±_ matrices, leading to a cost comparable to second-order MC-srDFT optimization. Such direct schemes can be straightforwardly adapted to the ERPA framework. Alternatively, one can employ an iterative algorithm for computing the ERPA response function presented in ref 62, leading to a fifth-power scaling. Finally, the computational cost of linear-response MC-srDFT can be further reduced by employing Cholesky decomposition of the two-electron integrals, as recently proposed for TD-MCSCF response by Nottoli, Lipparini, and coauthors.^65^

## 3 Performance of MC-srDFT Response for Polarizabilities

We assess the performance of MC-srDFT in predicting molecular polarizabilities and investigate the impact of tuning the RS parameter on the resulting accuracy. For comparison, we also present results obtained in two limiting cases of the parameter μ: μ = 0, where MC-srDFT reduces to KS-DFT and the linear response becomes equivalent to TD-DFT, and μ → ∞, where only the wave function component of MC-srDFT remains. If the latter is restricted to a single Slater determinant, the MC-srDFT response is identical to that of time-dependent Hartree–Fock (TD-HF). For genuinely multiconfigurational MC-srDFT, the response function χ^MC‑srDFT^ is obtained either from TD-MC-srDFT or from the ERPA-MC-srDFT approaches discussed in the previous section.

We employ a three-component naming convention to label the results: *Response-WF-srDF*, where the individual elements of the acronym denote the models used for the linear response, the wave function ansatz, and the short-range exchange-correlation density functional (srDF), respectively. For convenience, all acronyms introduced in this work, together with the corresponding regimes of the range-separation parameter, are summarized in Table 1.

**Table 1:** Response Models Considered in This Work Together with Their Acronyms^a^

Wave function	Short-range XC	Acronym
WFT limit (μ → ∞)		
HF	–	TD-HF
CASSCF	–	TD-CAS
CASSCF	–	ERPA-CAS
MC-srDFT (μ = 0.4 or opt)		
HF	srDF	TD-HF-srDF
CASSCF	srDF	TD-CAS-srDF
CASSCF	srDF	ERPA-CAS-srDF
DFT limit (μ = 0)		
–	DF	TD-DFT

a In cases where both TD-MC-srDFT and ERPA-MC-srDFT equations coincide, the same acronym applies. The short-range exchange-correlation (XC) functional (srDF) was taken as srLDA^56^ or srPBE^8^ (see Supporting Information for srPBE results).

The polarizability tensor is computed using the standard expression

26 $$\alpha_{ij}\left(\omega \right)=\underset{pqrs}{\sum }\chi_{pq,rs}^{\mathrm{MC‐srDFT}}\left(\omega \right)d_{rs}^{i}d_{pq}^{j}$$

where *i*, *j* = {*x*,*y*,*z*}, and $d_{rs}^{i},d_{pq}^{j}$ are dipole-moment matrix elements.

### 3.1 Computational Details

The test set comprises ground-state static and dynamic polarizabilities for 14 aromatic molecules reported in ref 52. Geometries optimized at the MP2/6-31G­(d) level for benzene, furan, pyrrole, imidazole, pyridine, pyrimidine, pyrazine, and pyridazine were taken from ref 30. The geometries of the remaining molecules were obtained at the same level of theory using the Molpro suite of programs.^66^

CASSCF reference wave functions were chosen as multiconfigurational wave functions in MC-srDFT. The MC-srDFT functional, eq 1, was optimized self-consistently using the DAlton program.^67^ The same software was used to perform TD-CAS, TD-HF, TD-DFT, and TD-CAS-srDFT response calculations. ERPA response calculations were carried out with the GammCor program,^68^ with one- and two-particle reduced density matrices corresponding to the CASSCF reference.

Two types of active spaces were employed. In TD-CAS and ERPA-CAS (the μ = ∞ limit), the active spaces were chosen based on information from the literature^30^ and physical insight. For finite RS parameters, we selected active spaces based on MP2-srDFT (μ = 0.4 bohr^–1^) natural occupations:^15^ all orbitals with occupation numbers <1.992 were considered active. Active spaces chosen this way are typically more compact compared to their μ = ∞ limit,^18^ and thus require fewer computational resources (notice that this is one of the advantages of MC-srDFT compared to the full-range multiconfigurational wave function methods). For comparison, MC-srDFT results obtained with the physically motivated CASSCF active spaces are also reported and marked with an asterisk (*). All employed active spaces are listed in Tables S1 and S2 in the Supporting Information.

The results presented in the main text were obtained with the LDA^69,70^ and srLDA^56^ functionals. In the Supporting Information, we also report results obtained with the srPBE^8^ model.

The RS parameter μ was tuned individually for each system. To this end, we performed a discrete scan of μ in the 0.20–0.50 bohr^–1^ range with a step of 0.05 bohr^–1^. For each value of μ, the largest (least negative) LR-EKT eigenvalue λ was extracted. The optimal μ was then determined by matching such obtained LR-EKT-based IPs to reference values using one-dimensional piecewise linear interpolation (see Figure 1 for an illustrative example of the benzene molecule and Table S3 in the Supporting Information). Reference IPs were calculated at the PBE0/aug-cc-pVTZ level of theory.

**Figure 1:**
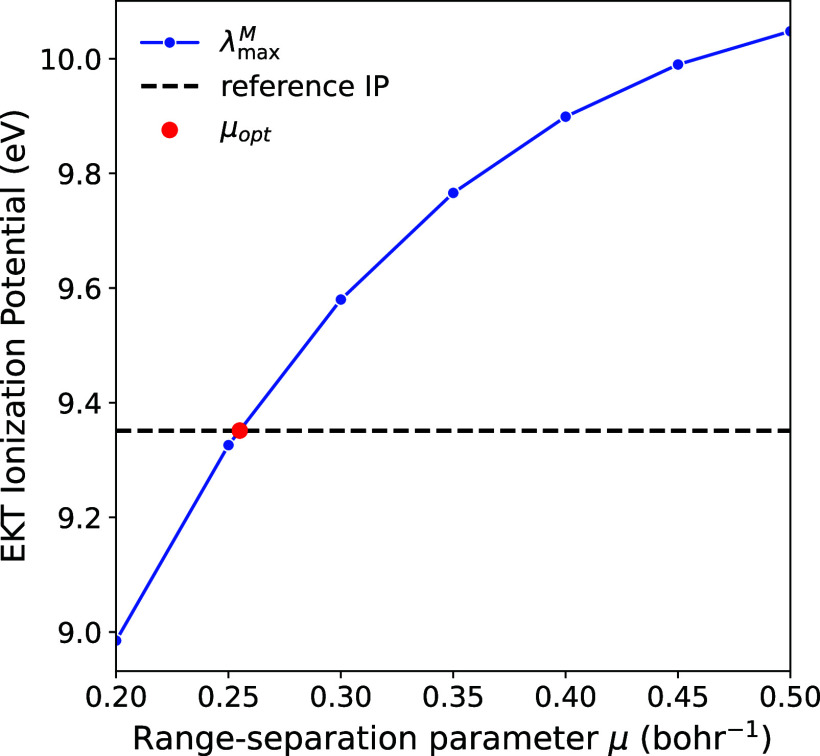
Tuning of the range-separation parameter μ for benzene. Blue dots denote the computed values of $\lambda_{\max}^{M}$ (largest eigenvalue of the EKT matrix) as a function of μ, while the solid blue line shows the piecewise-linear interpolation between these points. The dashed black line indicates the reference first ionization potential. The optimal value μ_opt_ (red marker) is determined as the intersection between the interpolated curve and the reference ionization potential.

All the polarizabilities were compared with reference CC3 ^71^ values taken from ref 52. The frequencies for the dynamic polarizabilities were identical to those used in ref 52, namely ω = 0.072003 au and 0.093215 au.

### 3.2 Results and Discussion

We begin by investigating the performance of linear-response-based methods which do not employ range separation, i.e., the μ = 0 and μ = ∞ limiting cases of MC-srDFT. The results are presented in Table 2. TD-CAS, ERPA-CASSCF, and TD-HF systematically underestimate polarizabilities, which can be attributed to the lack of dynamic correlation effects.^72,73^ In contrast, TD-LDA overestimates polarizabilities due to the erroneous asymptotic behavior of the LDA exchange-correlation potential.^45,46^ Somewhat surprisingly, TD-CAS performs markedly worse than TD-HF. This can be attributed to a cancellation of errors in TD-HF: it overestimates both excitation energies (see, e.g., ref 74) and transition dipole moments (cf. Figure S1 in the Supporting Information), leading to an overall reasonable accuracy of the polarizabilities. Replacing TD-HF with TD-CAS, even with relatively small active spaces, disrupts this fortuitous cancellation: the transition dipole moments are reduced, whereas the transition energies (i.e., the denominators in the response function) remain largely unchanged. As a result, the CASSCF polarizability components are smaller in magnitude than the corresponding HF values.

**Table 2:** Mean Error (ME), Mean Absolute Error (MAE), and Standard Deviation (STDEV) of the Errors in Static and Dynamic Polarizabilities (au) Computed with the aug-cc-pVTZ^75^ Basis Set

	TD-HF	TD-CAS	ERPA-CAS	TD-LDA
static				
MAE	0.97	3.63	4.82	2.30
ME	–0.97	–3.63	–4.82	2.30
STDEV	0.49	1.08	1.17	0.81
dynamic				
MAE	1.09	4.15	5.81	2.73
ME	–1.09	–4.15	–5.81	2.73
STDEV	0.57	1.64	1.52	1.03

A comparison of full TD-CAS and ERPA-CAS linear response shows that the former yields slightly more accurate results. The mean absolute errors (MAEs) for the static polarizabilities obtained with TD-CAS and ERPA-CAS amount to 3.6 au and 4.8 au, respectively. This indicates that the employed active spaces lead to sufficiently large CI expansions of the CASCI wave functions such that the response of the expansion coefficients, neglected in ERPA, becomes non-negligible.

The results for dynamic polarizabilities closely resemble those obtained for the static component (Table 2). Wave function-based approaches systematically underestimate the polarizabilities, with TD-HF providing the most accurate results. In contrast, the DFT-based method (TD-LDA) again overestimates the response. Although its accuracy remains inferior to TD-HF, it still outperforms the multiconfigurational methods considered here.

Next, we examine the performance of MC-srDFT with the standard value of the range-separation parameter, μ = 0.4 bohr^–1^ (see Table 3 and Figure 4). Increasing μ from 0 (the TD-LDA limit) to 0.4 bohr^–1^ reduces the errors in the polarizabilities for both linear response variants considered. While TD-LDA systematically overestimates the polarizabilities, CAS-srLDA underestimates them, indicating that μ = 0.4 bohr^–1^ already shifts the results toward the μ → ∞ limit, where the DFT component vanishes. The TD-CAS-srLDA and ERPA-CAS-srLDA approaches give polarizabilities of practically the same quality. This reflects the fact that compact active spaces in CAS-srLDA lead to short CI expansions of the CAS-srDFT wave function. Since static correlation effects are negligible, individual polarizabilities obtained from TD-CAS-srLDA and ERPA-CAS-srLDA calculations deviate from the HF-based values by no more than 0.3 au. Note that the inclusion of dynamic correlation in TD-HF-srLDA disrupts the error cancellation observed for TD-HF.

**Table 3:** Mean Error (ME), Mean Absolute Error (MAE), and Standard Deviation (STDEV) of the Errors in Static and Dynamic Polarizabilities (au) Obtained with Range-Separated Methods at *μ* = 0.4 bohr^–1^ Using the Aug-cc-pVTZ Basis Set^a^

	TD-HF-srLDA	TD-CAS-srLDA	ERPA-CAS-srLDA	TD-CAS*-srLDA	ERPA-CAS*-srLDA
static					
ME	–1.63	–1.70	–1.75	–1.67	–1.79
MAE	1.63	1.70	1.75	1.67	1.79
STDEV	0.54	0.53	0.56	0.57	0.55
MAX	–1.12	–1.16	–1.22	–1.02	–1.07
MIN	–2.93	–3.08	–3.22	–3.08	–3.18
dynamic					
ME	–1.78	–1.87	–1.95	–1.81	–2.00
MAE	1.78	1.87	1.95	1.81	2.00
STDEV	0.64	0.65	0.69	0.72	0.68
MAX	–1.17	–1.22	–1.29	–0.87	–1.12
MIN	–3.58	–3.87	–4.12	–3.87	–4.03

a CAS* indicates that a larger active space was employed, as described in Section 3.1.

Data in Table 3 indicates that there is only a marginal change in the polarizabilities when using the smaller (CAS) active spaces compared with the larger (CAS*) ones. This is expected since MC-srDFT by construction is less demanding when it comes to the length of the CI expansion than full-range MCSCF methods. Overall, this indicates that not only can the computationally cheaper ERPA approach be used without sacrificing accuracy, but a simpler wave function can also be employed, reducing both computational cost and convergence issues.

Even though range separation significantly improves the accuracy for all the CASSCF-based approaches relative to pure CASSCF-based response, the CAS-srDFT errors remain significant. The employment of the srPBE^76^ functional yields similar results, cf. Tables S4 and S5 in the Supporting Information. This indicates that the main source of error in the MC-srDFT response is likely the balance between the SR functional and the LR wave function; in other words, the chosen value of μ is not the most favorable.

We applied the optimal tuning of μ based on the exact condition in eq 17, obtaining μ values lying in quite a narrow interval, between 0.24 bohr^–1^ and 0.32 bohr^–1^ (see Figure 2 and Table S3 in the Supporting Information). Since the standard choice μ = 0.4 bohr^–1^ underestimates polarizabilities and TD-LDA (the μ = 0 bohr^–1^ limit) overestimates, the optimally tuned values are expected to improve the MC-srDFT predictions. Indeed, with the use of μ_opt_, the errors in all range-separated approaches decrease significantly, with MAE amounting to approximately 0.4 au for static polarizabilities (see Table 4) and to 0.5 au for dynamic polarizabilities (see Table 5). Additionally, the mean errors are close to zero for all three approaches, demonstrating that the optimally tuned μ_*opt*_ values effectively eliminate systematic over- or underestimation.

**Figure 2:**
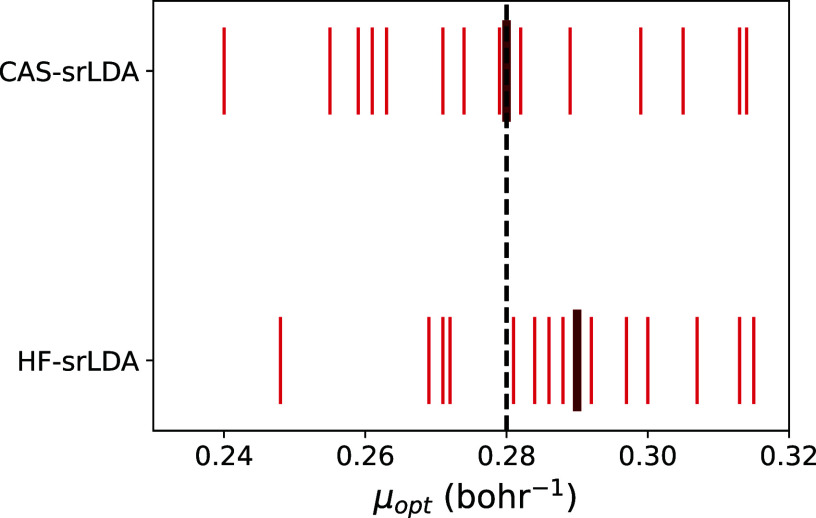
Distribution of the optimally tuned range-separation parameter μ*_opt_* for HF-srLDA and CAS-srLDA methods. Mean average values for each method are marked with dark-red lines and the average tuned value μ̅*_opt_* = 0.28 bohr^–1^ for CAS-srLDA is additionally marked with a dashed black line.

**Table 4:** Static (*ω* = 0) Polarizabilities (au) Compared with the Best Theoretical Estimate (CC3) and Experimental Values^a^ ^b^

	TD-HF-srLDA	TD-CAS-srLDA	ERPA-CAS-srLDA	CC3	Experiment
benzene	68.28	68.40	68.40	68.49	67.48 ^77^
benzonitrile	87.26	87.47	87.46	85.66	
furan	48.42	48.47	48.45	48.34	48.59 ^78^
imidazole	48.61	48.68	48.66	49.17	
oxazole	43.13	43.18	43.17	43.18	
phenol	74.71	74.83	74.82	74.15	
pyrazine	58.67	58.52	58.52	58.83	60.62 ^79^
pyridazine	58.51	58.45	58.43	58.73	59.32 ^79^
pyridine	63.24	63.23	63.23	63.19	64.11 ^79^
pyrimidine	57.82	57.78	57.78	57.81	59.35 ^79^
pyrrole	54.05	54.11	54.09	54.47	53.47 ^80^
phosphole	72.15	72.44	72.45	73.52	
thiazole	58.35	58.62	58.55	58.87	
thiophene	63.56	63.69	63.69	63.85	65.18 ^78^
ME	–0.11	–0.03	–0.04		
MAE	0.44	0.41	0.42		
STDEV	0.66	0.65	0.66		

a Mean error (ME), mean absolute error (MAE), and standard deviation (STDEV) of the errors for range-separated methods with tuned RS parameter *μ* obtained using the aug-cc-pVTZ basis set, calculated relative to CC3.

b Experimental results were taken from ref 77, ref 78, ref 79, ref 80, respectively.

**Table 5:** Dynamic Polarizabilities (au) Compared to the Best Theoretical Estimate (CC3)^a^

	TD-HF-srLDA	TD-CAS-srLDA	ERPA-CAS-srLDA	CC3
ω = 0.072003 au				
benzene	70.71	70.85	70.85	70.86
benzonitrile	90.90	91.14	91.13	88.94
furan	49.88	49.93	49.91	49.75
imidazole	50.04	50.12	50.09	50.65
oxazole	44.30	44.36	44.33	44.32
phenol	77.50	77.63	77.62	76.82
pyrazine	60.75	60.57	60.57	60.89
pyridazine	60.44	60.38	60.37	60.62
pyridine	65.40	65.39	65.38	65.31
pyrimidine	59.66	59.61	59.61	59.62
pyrrole	55.81	55.88	55.86	56.25
phosphole	75.28	75.59	75.58	76.85
thiazole	60.17	60.49	60.38	60.69
thiophene	65.71	65.84	65.84	65.98
ω = 0.093215 au				
benzene	72.54	72.69	72.69	72.63
benzonitrile	93.72	93.98	93.97	91.45
furan	50.97	51.02	51.00	50.80
imidazole	51.10	51.19	51.16	51.76
oxazole	45.16	45.23	45.20	45.16
phenol	79.63	79.77	79.77	78.85
pyrazine	62.38	62.17	62.16	62.50
pyridazine	61.94	61.89	61.88	62.07
pyridine	67.02	67.01	67.01	66.91
pyrimidine	61.04	60.99	60.99	60.98
pyrrole	57.13	57.20	57.18	57.59
phosphole	77.76	78.07	78.04	79.51
thiazole	61.53	61.90	61.74	62.05
thiophene	67.33	67.47	67.46	67.58
**ME**	–0.06	0.03	0.01	
**MAE**	0.51	0.48	0.49	
**STDEV**	0.81	0.81	0.82	

a Mean error (ME), mean absolute error (MAE), and standard deviation (STDEV) of the errors for range-separated methods with tuned RS parameter *μ* in the aug-cc-pVTZ basis set, calculated relative to CC3.

In Figure 3, we show the mean error as a function of the RS parameter. The minimum lies within the interval of the optimally tuned μ values. The average tuned parameter, μ̅_opt_ = 0.28 bohr^–1^, is close to the value that minimizes the mean error (ca. 0.25 bohr^–1^). Thus, we recommend using μ = 0.28 bohr^–1^ for ground-state polarizability calculations as a practical alternative to the more computationally demanding EKT-based tuning procedure.

**Figure 3:**
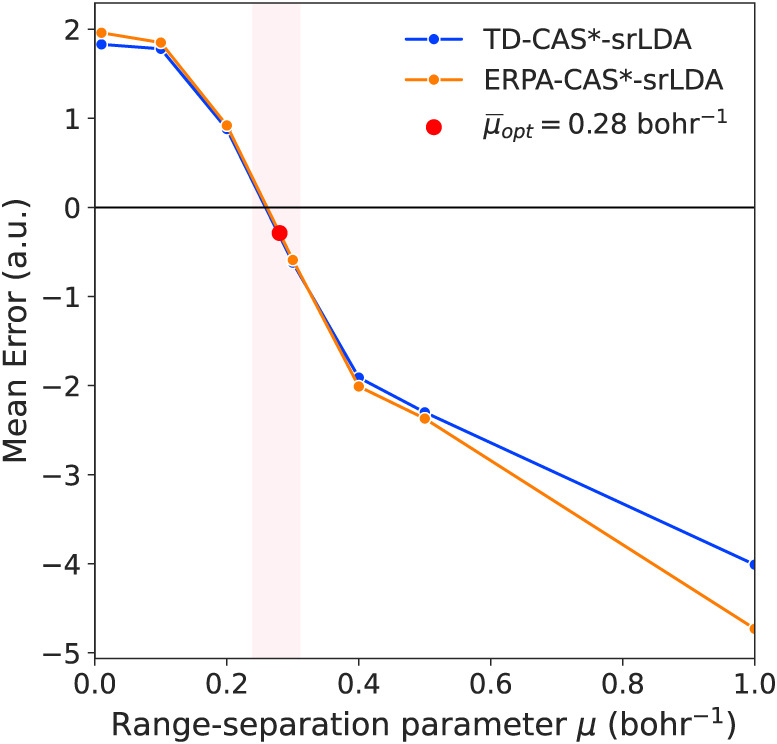
Mean error of static polarizabilities calculated in the aug-cc-pVDZ basis set. The area highlighted in red marks the range of tuned μ values for the studied systems, while the red dot marks the average tuned value μ̅_opt_ = 0.28 bohr^–1^. Pyridazine was excluded from the set due to poor convergence of the CASSCF wave function in this basis set. CAS* denotes that the larger active space was used, as described in Section 3.1.

Figure 4 shows the distribution of errors in a violin plot. For μ_opt_, unlike for μ = 0.4 bohr^–1^, the distributions of error for all three methods are approximately symmetric and unimodal, and the mean values are close to zero. The two outliers are benzonitrile and phosphole, the first having an error of 1.8 au for both TD-CAS-srLDA and ERPA-CAS-srLDA, and the second −1.1 au with optimally tuned values of μ_opt_ = 0.240 bohr^–1^ and μ_opt_ = 0.263 bohr^–1^, respectively. These errors are still within an acceptable range.

**Figure 4:**
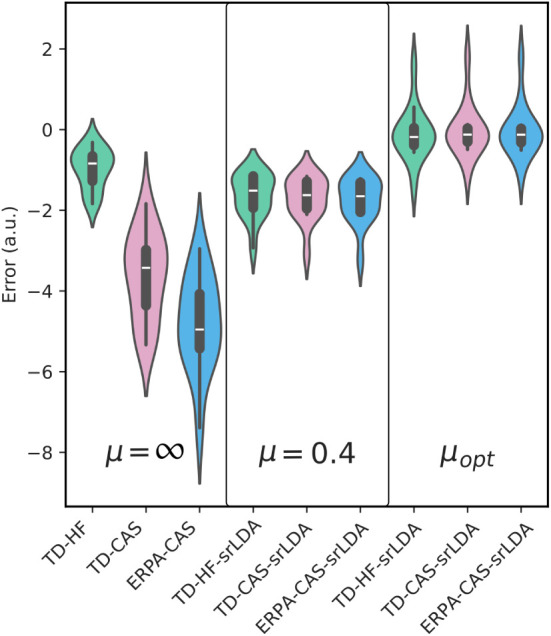
Violin plots of errors in static polarizability for TD-HF, TD-CAS, ERPA-CAS, and for the range-separated approaches with a standard value of the range-separation parameter (*μ* = 0.4 bohr^–1^) vs the optimally tuned values, μ*_opt_*.

## 4 Summary and Conclusions

We have proposed a range-separation parameter tuning procedure tailored to multiconfiguration short-range DFT. By generalizing the derivation of Morrell, Parr, and Levy,^58^ we have shown that the μ value can be determined to enforce the correct exponential decay of the approximate MC-srDFT electron density with the first ionization potential of a given system. Importantly, this ionization potential emerges as the largest (least negative) eigenvalue of the Extended Koopmans’ Theorem^59,60^ matrix constructed from the long-range Hamiltonian and the MC-srDFT reduced density matrices.

We have analyzed the performance of optimally tuned MC-srDFT employing CASSCF wave functions for static and dynamic polarizabilities of ground-state molecular systems taken from ref 52. Two variants were considered: the full linear-response formalism (TD-MC-srDFT)^13^ and its approximate counterpart based on the extended random phase approximation (ERPA-srDFT). Optimal tuning of CAS-srLDA leads to a clear improvement over the commonly used universal value μ = 0.4 bohr^–1^.^20^ The mean absolute TD-CAS-srLDA error with respect to the CC3 benchmark is reduced from 1.7 to 0.4 au and from 1.9 to 0.5 au for the static and dynamic polarizabilities, respectively. The srLDA and srPBE functionals exhibit similar performance, in agreement with previous observations for excitation energies^18^ and indirect spin–spin couplings.^31,32^ Since the optimally tuned μ values fall within a relatively narrow range, we recommend μ = 0.28 bohr^–1^ for ground-state polarizabilities as a practical alternative to system-specific tuning.

ERPA-srDFT gives results nearly identical to those obtained with the full TD-CAS-srDFT linear response. This stands in contrast to the comparison between the methods in the μ → ∞ limit, i.e., ERPA-CAS and TD-CAS, where ERPA is noticeably less accurate. The difference is readily understood: the inclusion of dynamical correlation via srDFT leads to more compact CI expansions, so that response contributions from the expansion coefficients become less important. Irrespective of the linear-response framework employed, MC-srDFT with the optimally tuned RS parameter greatly improves upon both of its μ limits, namely the systematic underestimation of polarizabilities by pure wave function approaches (TD-CAS and TD-HF) and the overestimation characteristic of TD-DFT.

Although the present study focuses on electronic ground states, the proposed EKT-based optimal-tuning MC-srDFT framework is readily extendable to excited states. Investigations in this direction are currently underway.

## Supplementary Material



## Data Availability

The data that support the findings of this study are available within the article and its Supporting Information. The raw data are available in the Zenodo repository at 10.5281/zenodo.19606039.

## Notes

Specification of the employed active spaces; comparison of TD-CAS and TD-HF transition dipole moment lengths; optimal values of the RS parameter; error statistics for polarizabilities obtained with the PBE and srPBE functionals; TD/ERPA-CAS-srDFT static and dynamic polarizabilities computed for μ = 0.4 bohr^–1^ and for the optimally tuned μ_opt_(srPBE) (PDF)

## References

[1] Hedegård, E. D. Quantum Chemistry and Dynamics of Excited States; John Wiley & Sons, Ltd, 2020, Vol. 3, pp. 47–75.

[2] Pernal K., Hapka M. (2022). Range-separated multiconfigurational
density functional theory methods. Wiley Interdiscip.
Rev.: Comput. Mol. Sci..

[3] Density Functional Methods in Physics, Dreizler, R. M. ; Providencia, J. Eds.; Springer: New York, 1985; pp. 177–207.

[4] Savin A., Flad H.-J. (1995). Density functionals for the Yukawa electron-electron
interaction. Int. J. Quantum Chem..

[5] Recent Developments of Modern Density Functional Theory, Seminario, J. M. ed.; Elsevier: Amsterdam, 1996; pp. 327–357.

[6] Leininger T., Stoll H., Werner H.-J., Savin A. (1997). Combining long-range
configuration interaction with short-range density functionals. Chem. Phys. Lett..

[7] Ferté A., Giner E., Toulouse J. (2019). Range-separated multideterminant
density-functional theory with a short-range correlation functional
of the on-top pair density. J. Chem. Phys..

[8] Goll E., Werner H.-J., Stoll H. (2005). A short-range gradient-corrected
density functional in long-range coupled-cluster calculations for
rare gas dimers. Phys. Chem. Chem. Phys..

[9] Goll E., Werner H.-J., Stoll H., Leininger T., Gori-Giorgi P., Savin A. (2006). A short-range gradient-corrected
spin density functional in combination with long-range coupled-cluster
methods: Application to alkali-metal rare-gas dimers. Chem. Phys..

[10] Fromager E., Cimiraglia R., Jensen H. J. Aa.. (2010). Merging multireference perturbation
and density-functional theories by means of range separation: Potential
curves for *Be*
_2_, *Mg*
_2_, and *Ca*
_2_. Phys. Rev. A.

[11] Hedegård E. D., Knecht S., Kielberg J. S., Jensen H. J. Aa., Reiher M. (2015). Density matrix
renormalization group with efficient dynamical electron correlation
through range separation. J. Chem. Phys..

[12] Hedegård E. D., Toulouse J., Jensen H. J. Aa. (2018). Multiconfigurational short-range
density-functional theory for open-shell systems. J. Chem. Phys..

[13] Fromager E., Knecht S., Jensen H. J. Aa. (2013). Multi-configuration
time-dependent
density-functional theory based on range separation. J. Chem. Phys..

[14] Pernal K. (2012). Excitation
energies from range-separated time-dependent density and density matrix
functional theory. J. Chem. Phys..

[15] Hubert M., Hedegård E. D., Jensen H. J. Aa. (2016). Investigation of Multiconfigurational
Short-Range Density Functional Theory for Electronic Excitations in
Organic Molecules. J. Chem. Theory Comput..

[16] Hubert M., Jensen H. J. Aa., Hedegård E. D. (2016). Excitation
Spectra of Nucleobases
with Multiconfigurational Density Functional Theory. J. Phys. Chem. A.

[17] Hedegård E. D. (2017). Assessment
of oscillator strengths with multiconfigurational short-range density
functional theory for electronic excitations in organic molecules. Mol. Phys..

[18] Kjellgren E. R., Hedegård E. D., Jensen H. J. Aa. (2019). Triplet excitation
energies from
multiconfigurational short-range density-functional theory response
calculations. J. Chem. Phys..

[19] Hapka M., Pastorczak E., Krzemińska A., Pernal K. (2020). Long-range-corrected
multiconfiguration density functional with the on-top pair density. J. Chem. Phys..

[20] Fromager E., Toulouse J., Jensen H. J. Aa. (2007). On
the universality of the long-/short-range
separation in multiconfigurational density-functional theory. J. Chem. Phys..

[21] Fromager E., Réal F., Wåhlin P., Wahlgren U., Jensen H. J. Aa. (2009). On
the universality of the long-/short-range separation in multiconfigurational
density-functional theory. II. Investigating f actinide species. J. Chem. Phys..

[22] Baer R., Livshits E., Salzner U. (2010). Tuned Range-Separated
Hybrids in
Density Functional Theory. Annu. Rev. Phys.
Chem..

[23] Kronik L., Stein T., Refaely-Abramson S., Baer R. (2012). Excitation Gaps of
Finite-Sized Systems from Optimally Tuned Range-Separated Hybrid Functionals. J. Chem. Theory Comput..

[24] Autschbach J., Srebro M. (2014). Delocalization Error and “Functional Tuning”
in Kohn-Sham Calculations of Molecular Properties. Acc. Chem. Res..

[25] Levy M., Perdew J. P., Sahni V. (1984). Exact differential equation for the
density and ionization energy of a many-particle system. Phys. Rev. A.

[26] Almbladh C.-O., von Barth U. (1985). Exact results
for the charge and spin densities, exchange-correlation
potentials, and density-functional eigenvalues. Phys. Rev. B.

[27] Modrzejewski M., Rajchel Ł., Chałasiński G., Szczęśniak M. M. (2013). Density-Dependent
Onset of the Long-Range Exchange: A Key to Donor-Acceptor Properties. J. Phys. Chem. A.

[28] Mandal A., Herbert J. M. (2025). Simplified Tuning of Long-Range Corrected Time-Dependent
Density Functional Theory. J. Phys. Chem. Lett..

[29] Pernal K., Chatterjee K., Kowalski P. H. (2014). How accurate is the strongly orthogonal
geminal theory in predicting excitation energies? Comparison of the
extended random phase approximation and the linear response theory
approaches. J. Chem. Phys..

[30] Schreiber M., Silva-Junior M. R., Sauer S. P. A., Thiel W. (2008). Benchmarks for electronically
excited states: CASPT2, CC2, CCSD, and CC3. J. Chem. Phys..

[31] Kjellgren E. R., Jensen H. J. Aa. (2021). Multi-configurational
short-range density functional
theory can describe spin-spin coupling constants of transition metal
complexes. J. Chem. Phys..

[32] Hapka M., Jensen H. J. Aa. (2025). Time-Dependent
Multiconfigurational Short-Range Density
Functional Theory with Generalized Valence Bond Wave Functions. J. Phys. Chem. A.

[33] Hait D., Head-Gordon M. (2018). How accurate are static polarizability
predictions
from density functional theory? An assessment over 132 species at
equilibrium geometry. Phys. Chem. Chem. Phys..

[34] Wilkins D. M., Grisafi A., Yang Y., Lao K. U., DiStasio R. A., Ceriotti M. (2019). Accurate molecular
polarizabilities
with coupled cluster theory and machine learning. Proc. Natl. Acad. Sci. U. S. A..

[35] Sun H., Autschbach J. (2013). Influence
of the Delocalization Error and Applicability
of Optimal Functional Tuning in Density Functional Calculations of
Nonlinear Optical Properties of Organic Donor-Acceptor Chromophores. ChemPhysChem.

[36] Nenon S., Champagne B., Spassova M. I. (2014). Assessing long-range corrected functionals
with physically-adjusted range-separated parameters for calculating
the polarizability and the second hyperpolarizability of polydiacetylene
and polybutatriene chains. Phys. Chem. Chem.
Phys..

[37] Oviedo M.
B., Ilawe N. V., Wong B. M. (2016). Polarizabilities of *π*-Conjugated
Chains Revisited: Improved Results from Broken-Symmetry
Range-Separated DFT and New CCSD­(T) Benchmarks. J. Chem. Theory Comput..

[38] Alipour M., Fallahzadeh P. (2017). Nonempirically tuning range-separated
functionals for
dipole polarizabilities of nanostructures containing hydrogen bonds. Theor. Chem. Acc..

[39] Garrett K., Sosa Vazquez X., Egri S. B., Wilmer J., Johnson L. E., Robinson B. H., Isborn C. M. (2014). Optimum Exchange for Calculation
of Excitation Energies and Hyperpolarizabilities of Organic Electro-optic
Chromophores. J. Chem. Theory Comput..

[40] Garza A. J., Osman O. I., Asiri A. M., Scuseria G. E. (2015). Can Gap Tuning Schemes
of Long-Range Corrected Hybrid Functionals Improve the Description
of Hyperpolarizabilities?. J. Phys. Chem. B.

[41] Wang C., Yuan Y., Tian X. (2017). Assessment
of range-separated exchange
functionals and nonempirical functional tuning for calculating the
static second hyperpolarizabilities of streptocyanines. J. Comput. Chem..

[42] Zaleśny R., Medved’ M., Sitkiewicz S. P., Matito E., Luis J. M. (2019). Can Density
Functional Theory Be Trusted for High-Order Electric Properties? The
Case of Hydrogen-Bonded Complexes. J. Chem.
Theory Comput..

[43] Lescos L., Sitkiewicz S. P., Beaujean P., Blanchard-Desce M., Champagne B., Matito E., Castet F. (2020). Performance of DFT
functionals for calculating the second-order nonlinear optical properties
of dipolar merocyanines. Phys. Chem. Chem. Phys..

[44] Besalu-Sala P., Sitkiewicz S. P., Salvador P., Matito E., Luis J. M. (2020). A new tuned
range-separated density functional for the accurate calculation of
second hyperpolarizabilities. Phys. Chem. Chem.
Phys..

[45] Tozer D. J., Handy N. C. (1998). Improving virtual
Kohn-Sham orbitals and eigenvalues:
Application to excitation energies and static polarizabilities. J. Chem. Phys..

[46] Grüning M., Gritsenko O. V., van Gisbergen S. J. A., Baerends E. J. (2001). Shape corrections
to exchange-correlation potentials by gradient-regulated seamless
connection of model potentials for inner and outer region. J. Chem. Phys..

[47] Cencek W., Szalewicz K. (2013). On asymptotic behavior of density functional theory. J. Chem. Phys..

[48] Jansen G. (2014). Symmetry-adapted
perturbation theory based on density functional theory for noncovalent
interactions. Wiley Interdiscip. Rev.: Comput.
Mol. Sci..

[49] Patkowski K. (2020). Recent developments
in symmetry-adapted perturbation theory. Wiley
Interdiscip. Rev.: Comput. Mol. Sci..

[50] Chatterjee K., Pernal K. (2012). Excitation energies from extended random phase approximation
employed with approximate one-and two-electron reduced density matrices. J. Chem. Phys..

[51] Drwal D., Pastorczak E., Pernal K. (2021). Excited states in the adiabatic connection
fluctuation-dissipation theory: Recovering missing correlation energy
from the negative part of the density response spectrum. J. Chem. Phys..

[52] Jørgensen M. W., Faber R., Ligabue A., Sauer S. P. A. (2020). Benchmarking
Correlated Methods for Frequency-Dependent Polarizabilities: Aromatic
Molecules with the CC3, CCSD, CC2, SOPPA, SOPPA­(CC2), and SOPPA­(CCSD)
Methods. J. Chem. Theory Comput..

[53] Pollet R., Savin A., Leininger T., Stoll H. (2002). Combining multideterminantal
wave functions with density functionals to handle near-degeneracy
in atoms and molecules. J. Chem. Phys..

[54] Toulouse J., Colonna F., Savin A. (2004). Long-range–short-range
separation
of the electron-electron interaction in density-functional theory. Phys. Rev. A.

[55] Toulouse J., Gori-Giorgi P., Savin A. (2005). A short-range correlation energy
density functional with multi-determinantal reference. Theor. Chem. Acc..

[56] Paziani S., Moroni S., Gori-Giorgi P., Bachelet G. B. (2006). Local-spin-density
functional for multideterminant density functional theory. Phys. Rev. B.

[57] Goll E., Ernst M., Moegle-Hofacker F., Stoll H. (2009). Development and assessment
of a short-range meta-GGA functional. J. Chem.
Phys..

[58] Morrell M. M., Parr R. G., Levy M. (1975). Calculation of ionization potentials
from density matrices and natural functions, and the long-range behavior
of natural orbitals and electron density. J.
Chem. Phys..

[59] Morrison R.
C. (1992). The extended
Koopmans’ theorem and its exactness. J. Chem. Phys..

[60] Smith D. W., Day O. W. (1975). Extension of Koopmans’ theorem. I. derivation. J. Chem. Phys..

[61] Rowe D. J. (1968). Equations-of-Motion
Method and the Extended Shell Model. Rev. Mod.
Phys..

[62] Drwal D., Beran P., Hapka M., Modrzejewski M., Sokół A., Veis L., Pernal K. (2022). Efficient
adiabatic
connection approach for strongly correlated systems: Application to
singlet–triplet gaps of biradicals. J.
Phys. Chem. Lett..

[63] Jørgensen P., Jensen H. J. Aa., Olsen J. (1988). Linear response calculations for
large scale multiconfiguration self-consistent field wave functions. J. Chem. Phys..

[64] Olsen J., Jensen H. J. Aa., Jørgensen P. (1988). Solution of
the large matrix equations
which occur in response theory. J. Comput. Phys..

[65] Nottoli T., Lapi L., Alessandro R., Gianní I., Pes F., Lipparini F. (2025). An Efficient
and Robust Implementation of CASSCF Linear
Response Theory. J. Phys. Chem. A.

[66] Werner H.-J., Knowles P. J., Knizia G., Manby F. R., Schütz M. (2012). Molpro: a
general-purpose quantum chemistry program package. Wiley Interdiscip. Rev. Comput. Mol. Sci..

[67] Aidas K., Angeli C., Bak K. L., Bakken V., Bast R., Boman L., Christiansen O., Cimiraglia R., Coriani S., Dahle P., Dalskov E. K., Ekström U., Enevoldsen T., Eriksen J. J., Ettenhuber P., Fernández B., Ferrighi L., Fliegl H., Frediani L., Hald K., Halkier A., Hättig C., Heiberg H., Helgaker T., Hennum A. C., Hettema H., Hjertenæs E., Høst S., Høyvik I.-M., Iozzi M. F., Jansík B., Jensen H. J. Aa., Jonsson D., Jørgensen P., Kauczor J., Kirpekar S., Kjærgaard T., Klopper W., Knecht S., Kobayashi R., Koch H., Kongsted J., Krapp A., Kristensen K., Ligabue A., Lutnæs O. B., Melo J. I., Mikkelsen K. V., Myhre R. H., Neiss C., Nielsen C. B., Norman P., Olsen J., Olsen J. M. H., Osted A., Packer M. J., Pawlowski F., Pedersen T. B., Provasi P. F., Reine S., Rinkevicius Z., Ruden T. A., Ruud K., Rybkin V. V., Sałek P., Samson C. C. M., de Merás A. S., Saue T., Sauer S. P. A., Schimmelpfennig B., Sneskov K., Steindal A. H., Sylvester-Hvid K. O., Taylor P. R., Teale A. M., Tellgren E. I., Tew D. P., Thorvaldsen A. J., Thøgersen L., Vahtras O., Watson M. A., Wilson D. J. D., Ziolkowski M., Ågren H. (2014). The Dalton
quantum chemistry program system. WIREs Comput.
Mol. Sci..

[68] Pernal, K. ; Hapka, M. ; Przybytek, M. ; Modrzejewski, M. ; Sokół, A. GammCor code; https://github.com/pernalk/GAMMCOR, accessed April 26, 2026.

[69] Slater, J. C. Quantum Theory of Molecular and Solids. In Self-Consistent Field for Molecular and Solids; McGraw-Hill, 1974; Vol. 4.

[70] Vosko S. H., Wilk L., Nusair M. (1980). Accurate spin-dependent electron
liquid correlation energies for local spin density calculations: a
critical analysis. Can. J. Phys..

[71] Christiansen O., Koch H., Jørgensen P. (1995). Response functions
in the CC3 iterative
triple excitation model. J. Chem. Phys..

[72] Parasuk V., Neogrády P., Lischka H., Urban M. (1996). A Comparison of Variational
and Coupled-Cluster Calculations of Molecular Properties: The Polarizabilities
of BeO, ^1^Σ_g_
^+^ and C_2_, ^1^Σ_g_
^+^, ^3^Π_u_, and ^3^Σ_g_
^–^. J. Phys. Chem..

[73] Champagne B., Botek E., Nakano M., Nitta T., Yamaguchi K. (2005). Basis set
and electron correlation effects on the polarizability and second
hyperpolarizability of model open-shell *π*-conjugated
systems. J. Chem. Phys..

[74] Hapka M., Pernal K., Jensen H. J. Aa. (2022). An
efficient implementation of time-dependent
linear-response theory for strongly orthogonal geminal wave function
models. J. Chem. Phys..

[75] Kendall R.
A., Dunning T. H., Harrison R. J. (1992). Electron affinities
of the first-row atoms revisited. Systematic basis sets and wave functions. J. Chem. Phys..

[76] Perdew J. P., Burke K., Ernzerhof M. (1996). Generalized Gradient Approximation
Made Simple. Phys. Rev. Lett..

[77] Alms G. R., Burnham A., Flygare W. H. (1975). Measurement
of the dispersion in
polarizability anisotropies. J. Chem. Phys..

[78] Kamada K., Ueda M., Nagao H., Tawa K., Sugino T., Shmizu Y., Ohta K. (2000). Molecular
Design for Organic Nonlinear
Optics: Polarizability and Hyperpolarizabilities of Furan Homologues
Investigated by Ab Initio Molecular Orbital Method. J. Phys. Chem. A.

[79] Soscun H., Bermudez Y., Castellano O., Hernandez J. (2004). Effects of
protonation on the dipole polarizability of monocyclic azines: a theoretical
study. Chem. Phys. Lett..

[80] Hinchliffe A., Soscún M. H. J. (1995). Ab initio
studies of the dipole polarizabilities of
conjugated molecules: Part 5. The five-membered heterocyclics C_4_H_4_E (E = BH, AlH, CH_2_, SiH_2_, NH, PH, O and S). J. Mol. Struct..

