# Supplementary material for: Optimally Tuned Multiconfigurational Short-Range DFT for Linear Response Properties
Source: J Phys Chem A. 2026 May 8;130(20):3996–4006. doi: 10.1021/acs.jpca.6c01551 (PMC13308998; doi:10.1021/acs.jpca.6c01551)
Supplement: Supplementary file 1 [file jp6c01551_si_001.pdf]

Supporting Information: Optimally Tuned Multiconfigurational Short-Range DFT for Linear Response Properties

Michał Hapka,*,† Katarzyna Pernal,‡ and Ewa Pastorczak*,‡

†*University of Warsaw, Faculty of Chemistry, ul. L. Pasteura 1, 02-093 Warsaw, Poland*

‡*Institute of Physics, Lodz University of Technology, ul. Wolczanska 217/221, 93-005 Lodz, Poland*

E-mail: michal.hapka@gmail.com; ewapastorczak@gmail.com

Table S1: CAS-srDFT ($\mu = 0.4$) active space.

Molecule	Used symmetry	Inactive	CAS	Electrons
benzene	D_{2h}	6 5 4 3 1 0 0 0	0 0 0 0 1 1 1 1	4
benzonitrile	C_{2v}	15 1 7 0	0 4 2 2	8
furan	C_{2v}	9 1 6 0	0 2 0 2	4
imidazole	C_s	15 1	0 4	4
oxazole	C_s	15 1	0 4	4
phenol	C_s	21 2	0 4	4
pyrazine	D_{2h}	6 1 5 0 4 0 3 0	0 1 0 1 0 1 0 1	4
pyridazine	C_{2v}	10 1 8 0	0 2 0 2	4
pyridine	C_{2v}	11 1 7 0	0 2 0 2	4
pyrimidine	C_{2v}	11 1 7 0	0 2 0 2	4
pyrrole	C_{2v}	9 1 6 0	0 2 0 2	4
phospholе	C_{2v}	10 2 6 0	2 2 2 2	8
thiazole	C_s	16 2	4 4	8
thiophene	C_{2v}	10 2 6 0	2 2 2 2	8

Table S2: The $\mu = \infty$ active space used in calculations (results marked with an asterisk, CAS*).

Molecule	Used symmetry	Inactive	CAS	Electrons
benzene	D_{2h}	6 5 4 3 0 0 0 0	0 0 0 0 2 1 2 1	6
benzonitrile	C_{2v}	12 0 6 0	4 5 3 2	18
furan	C_{2v}	9 0 6 0	0 3 0 2	6
imidazole	C_s	14 0	1 6	8
oxazole	C_s	10 2	7 3	12
phenol	C_s	20 1	2 6	8
pyrazine	D_{2h}	5 0 4 0 4 0 3 0	1 2 1 1 0 2 0 1	10
pyridazine	C_{2v}	9 0 5 0	3 4 3 3	14
pyridine	C_{2v}	10 0 7 0	1 4 0 2	8
pyrimidine	C_{2v}	10 0 6 0	1 4 2 2	10
pyrrole	C_{2v}	9 0 6 0	0 3 0 2	6
phosphole	C_{2v}	10 2 7 0	2 2 0 2	6
thiazole	C_s	15 2	6 4	10
thiophene	C_{2v}	11 1 7 0	1 3 0 2	6

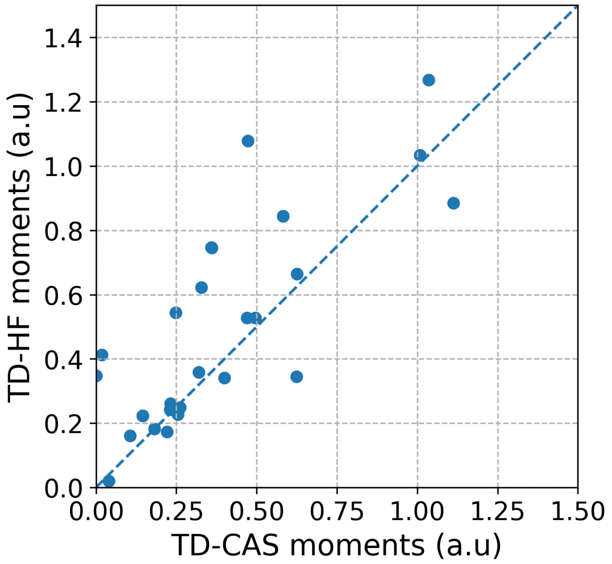

Figure S1: Transition dipole moments for the lowest allowed transitions.

Table S3: Optimally-tuned μ_{opt} values obtained in the aug-cc-pVDZ basis set.

molecule	HF-srLDA	CAS-srLDA	HF-srPBE	CAS-srPBE
benzene	0.269	0.255	0.290	0.274
benzonitrile	0.248	0.240	0.271	0.271
furan	0.288	0.279	0.310	0.297
imidazole	0.292	0.282	0.316	0.301
oxazole	0.297	0.289	0.324	0.311
phenol	0.272	0.259	0.291	0.277
phosphole	0.286	0.263	0.303	0.278
pyrazine	0.313	0.313	0.316	0.316
pyridazine	0.315	0.314	0.301	0.301
pyridine	0.300	0.299	0.314	0.313
pyrimidine	0.307	0.305	0.312	0.311
pyrrole	0.284	0.274	0.302	0.291
thiazole	0.281	0.271	0.301	0.288
thiophene	0.271	0.261	0.289	0.278

Table S4: Error statistics for static and dynamic polarizabilities (a.u.) obtained with range-separated methods at $\mu = 0.4$ bohr^{-1} using the aug-cc-pVTZ basis set. CAS-srPBE results marked with an asterisk correspond to $\mu = \infty$ active space (see Table S2).

static	TD-PBE	TD-HF -srPBE	TD-CAS -srPBE	ERPA-CAS -srPBE	TD-CAS* -srPBE	ERPA-CAS* -srPBE
ME	2.10	−1.30	−1.37	−1.60	−1.34	−1.64
MAE	2.10	1.30	1.37	1.60	1.34	1.64
SD	0.78	0.52	0.52	0.54	0.56	0.53
MAX	4.38	−0.82	−0.88	−1.08	−0.68	−0.94
MIN	1.25	−2.55	−2.70	−3.01	−2.70	−2.97
dynamic						
ME	2.54	−1.42	−1.51	−1.78	−1.44	−1.83
MAE	2.54	1.42	1.51	1.78	1.44	1.83
SD	1.01	0.62	0.63	0.66	0.71	0.65
MAX	5.97	−0.79	−0.80	−1.14	−0.49	−0.98
MIN	1.40	−3.13	−3.42	−3.86	−3.42	−3.76

Table S5: Error statistics for static and dynamic polarizabilities (a.u.) obtained with range-separated methods at $\mu = 0.4$ bohr^{-1} using the aug-cc-pVTZ basis set. CAS-srLDA results marked with an asterisk are obtained in a larger active space. Basis set is **aug-cc-pVDZ**.

static	TD-LDA	TD-HF-<br>-srLDA	TD-CAS<br>-srLDA	ERPA-CAS<br>-srLDA	TD-CAS*<br>-srLDA	ERPA-CAS*<br>-srLDA
ME	2.06	−1.85	−1.92	−1.98	−2.82	−2.02
MAE	2.06	1.85	1.92	1.98	2.82	2.02
SD	0.82	0.61	0.59	0.62	3.34	0.61
MAX	4.35	−1.27	−1.35	−1.37	−1.22	−1.28
MIN	1.21	−3.29	−3.42	−3.59	−14.22	−3.55
dynamic						
ME	2.46	−2.04	−2.13	−2.21	−2.15	−2.26
MAE	2.46	2.04	2.13	2.21	2.15	2.26
SD	1.06	0.72	0.73	0.77	0.75	0.75
MAX	5.90	−1.35	−1.43	−1.46	−1.27	−1.34
MIN	1.27	−4.07	−4.37	−4.62	−4.36	−4.53

Table S6: Error statistics for static and dynamic polarizabilities. Range separation parameter $\mu = 0.4$ bohr^{-1}. **CAS-srPBE** results marked with an asterisk are obtained in a larger active space. Basis set is **aug-cc-pVDZ**.

static	TD-PBE	TD-HF-<br>-srPBE	TD-CAS<br>-srPBE	ERPA-CAS<br>-srPBE	TD-CAS*<br>-srPBE	ERPA-CAS*<br>-srPBE
ME	1.79	−1.56	−1.63	−1.87	−1.51	−1.91
MAE	1.79	1.56	1.63	1.87	1.54	1.91
SD	0.80	0.59	0.57	0.60	0.77	0.59
MAX	4.01	−0.99	−1.10	−1.30	0.19	−1.18
MIN	0.75	−2.95	−3.09	−3.43	−3.11	−3.39
dynamic						
ME	2.18	−1.71	−1.81	−2.09	−2.10	−2.14
MAE	2.18	1.71	1.81	2.09	2.10	2.14
SD	1.03	0.70	0.71	0.74	1.18	0.73
MAX	5.53	−1.05	−1.17	−1.40	−0.91	−1.24
MIN	0.80	−3.67	−3.98	−4.42	−6.23	−4.33

Table S7: Static ($\omega = 0$) polarizabilities (a.u.) compared to the best theoretical estimate (CC3) and experimental values. Mean error (ME), mean absolute error (MAE), and standard deviation (SD) of the errors for range-separated methods at $\mu = 0.4\,\text{bohr}^{-1}$ obtained using the aug-cc-pVTZ basis set, calculated relative to CC3. Experimental results were taken from [a]Ref. S1, [b]Ref. S2, [c]Ref. S3, [d]Ref. S4, respectively.

	TD-HF-srLDA	TD-CAS-srLDA	ERPA-CAS-srLDA	CC3	Experiment
benzene	66.54	66.39	66.38	68.49	67.48[a]
benzonitrile	84.34	84.29	84.24	85.66	
furan	47.19	47.10	47.04	48.34	48.59[b]
imidazole	47.27	47.23	47.16	49.17	
oxazole	42.06	42.02	41.96	43.18	
phenol	72.45	72.28	72.27	74.15	
pyrazine	57.67	57.47	57.46	58.83	60.62[c]
pyridazine	57.48	57.40	57.38	58.73	59.32[c]
pyridine	62.05	61.92	61.91	63.19	64.11[c]
pyrimidine	56.66	56.60	56.59	57.81	59.35[c]
pyrrole	52.58	52.51	52.43	54.47	53.47[d]
phosphole	70.59	70.44	70.30	73.52	
thiazole	56.73	56.93	56.82	58.87	
thiophene	61.83	61.86	61.81	63.85	65.18[b]
**ME**	−1.63	−1.70	−1.75		
**MAE**	1.63	1.70	1.75		
**SD**	0.54	0.53	0.56		

Table S8: Dynamic polarizabilities (a.u.) compared to the best theoretical estimate (CC3). Mean error (ME), mean absolute error (MAE), and standard deviation (SD) of the errors for range-separated methods at $\mu = 0.4\,\mathrm{bohr}^{-1}$, calculated relative to CC3. The basis set is aug-cc-pVTZ.

	TD-HF-srLDA	TD-CAS-srLDA	ERPA-CAS-srLDA	CC3
$\omega = \mathbf{0.072003}$ a.u.				
benzene	68.80	68.64	68.63	70.86
benzonitrile	87.62	87.58	87.46	88.94
furan	48.54	48.44	48.37	49.75
imidazole	48.59	48.55	48.46	50.65
oxazole	43.15	43.10	43.03	44.32
phenol	75.00	74.81	74.80	76.82
pyrazine	59.68	59.43	59.42	60.89
pyridazine	59.31	59.24	59.21	60.62
pyridine	64.10	63.96	63.94	65.31
pyrimidine	58.40	58.33	58.32	59.62
pyrrole	54.21	54.12	54.02	56.25
phosphole	73.58	73.36	73.16	76.85
thiazole	58.42	58.63	58.49	60.69
thiophene	63.82	63.84	63.78	65.98
$\omega = \mathbf{0.093215}$ a.u.				
benzene	70.50	70.32	70.31	72.63
benzonitrile	90.13	90.10	89.93	91.45
furan	49.55	49.44	49.36	50.80
imidazole	49.56	49.52	49.42	51.76
oxazole	43.95	43.91	43.82	45.16
phenol	76.93	76.72	76.71	78.85
pyrazine	61.25	60.94	60.93	62.50
pyridazine	60.72	60.66	60.63	62.07
pyridine	65.65	65.49	65.47	66.91
pyrimidine	59.70	59.62	59.61	60.98
pyrrole	55.42	55.32	55.21	57.59
phosphole	75.93	75.64	75.39	79.51
thiazole	59.67	59.89	59.73	62.05
thiophene	65.32	65.33	65.25	67.58
**ME**	−1.74	−1.82	−1.89	
**MAE**	1.74	1.82	1.89	
**SD**	0.60	0.61	0.64	

Table S9: Static ($\omega = 0$) polarizabilities (a.u.) computed with TD/ERPA-WF-srPBE compared to the CC3 reference and experimental values. Mean error (ME), mean absolute error (MAE), and standard deviation (SD) of the errors at $\mu = 0.4\,\text{bohr}^{-1}$, calculated relative to CC3. The basis set is aug-cc-pVTZ.

	TD-HF-srPBE	TD-CAS-srPBE	ERPA-CAS-srPBE	CC3	Experiment
benzene	66.87	66.72	66.51	68.49	67.48[a]
benzonitrile	84.81	84.78	84.46	85.66	
furan	47.49	47.41	47.20	48.34	48.59[b]
imidazole	47.57	47.54	47.32	49.17	
oxazole	42.33	42.29	42.10	43.18	
phenol	72.83	72.66	72.43	74.15	
pyrazine	57.97	57.77	57.57	58.83	60.62[c]
pyridazine	57.78	57.70	57.49	58.73	59.32[c]
pyridine	62.37	62.24	62.03	63.19	64.11[c]
pyrimidine	56.95	56.89	56.70	57.81	59.35[c]
pyrrole	52.92	52.84	52.60	54.47	53.47[d]
phosphole	70.97	70.82	70.51	73.52	
thiazole	57.05	57.25	57.00	58.87	
thiophene	62.17	62.20	61.99	63.85	65.18[b]
**ME**	−1.30	−1.37	−1.60		
**MAE**	1.30	1.37	1.60		
**SD**	0.54	0.52	0.54		

Table S10: Dynamic polarizabilities (a.u.) computed with TD/ERPA-WF-srPBE compared to CC3 results. Mean error (ME), mean absolute error (MAE), and standard deviation (SD) of the errors obtained with RS methods at $\mu = 0.4\,\mathrm{bohr}^{-1}$, calculated relative to CC3. The basis set is aug-cc-pVTZ.

	TD-HF-srPBE	TD-CAS-srPBE	ERPA-CAS-srPBE	CC3
$\omega = \mathbf{0.072003}$ a.u.				
benzene	69.17	69.00	68.78	70.86
benzonitrile	88.12	88.10	87.69	88.94
furan	48.87	48.77	48.54	49.75
imidazole	48.91	48.87	48.63	50.65
oxazole	43.44	43.39	43.18	44.32
phenol	75.40	75.21	74.96	76.82
pyrazine	59.99	59.74	59.52	60.89
pyridazine	59.62	59.55	59.32	60.62
pyridine	64.44	64.29	64.07	65.31
pyrimidine	58.70	58.63	58.43	59.62
pyrrole	54.57	54.48	54.22	56.25
phosphole	74.00	73.78	73.41	76.85
thiazole	58.76	58.98	58.68	60.69
thiophene	64.19	64.20	63.97	65.98
$\omega = \mathbf{0.093215}$ a.u.				
benzene	70.88	70.70	70.47	72.63
benzonitrile	90.66	90.65	90.17	91.45
furan	49.90	49.79	49.54	50.80
imidazole	49.91	49.86	49.60	51.76
oxazole	44.26	44.21	43.97	45.16
phenol	77.36	77.14	76.88	78.85
pyrazine	61.57	61.26	61.03	62.50
pyridazine	61.04	60.96	60.72	62.07
pyridine	66.00	65.83	65.60	66.91
pyrimidine	60.01	59.93	59.72	60.98
pyrrole	55.81	55.70	55.42	57.59
phosphole	76.38	76.10	75.65	79.51
thiazole	60.03	60.26	59.93	62.05
thiophene	65.71	65.71	65.46	67.58
**ME**	−1.42	−1.51	−1.78	
**MAE**	1.42	1.51	1.78	
**SD**	0.62	0.63	0.66	

Table S11: Dynamic polarizabilities (a.u.) compared to the CC3 reference. Mean error (ME), mean absolute error (MAE), and standard deviation (SD) of the errors for range-separated methods with tuned RS parameter μ_{opt} in the aug-cc-pVTZ basis set.

	TD-HF-srPBE	TD-CAS-srPBE	ERPA-CAS-srPBE	CC3
$\omega = \mathbf{0.072003}$ a.u.				
benzene	70.66	70.82	70.44	70.86
benzonitrile	90.74	90.90	90.37	88.94
furan	49.89	49.99	49.71	49.75
imidazole	49.99	50.14	49.86	50.65
oxazole	44.27	44.36	44.11	44.32
phenol	77.39	77.56	77.14	76.82
pyrazine	60.99	60.82	60.52	60.89
pyridazine	60.71	60.67	60.37	60.62
pyridine	65.70	65.67	65.32	65.31
pyrimidine	59.85	59.80	59.51	59.62
pyrrole	55.83	55.94	55.63	56.25
phosphole	75.32	75.68	75.30	76.85
thiazole	60.12	60.51	60.13	60.69
thiophene	65.70	65.88	65.55	65.98
$\omega = \mathbf{0.093215}$ a.u.				
benzene	72.49	72.65	72.25	72.63
benzonitrile	93.53	93.73	93.12	91.45
furan	50.98	51.08	50.79	50.80
imidazole	51.05	51.21	50.91	51.76
oxazole	45.13	45.23	44.97	45.16
phenol	79.51	79.69	79.25	78.85
pyrazine	62.62	62.42	62.10	62.50
pyridazine	62.20	62.18	61.86	62.07
pyridine	67.34	67.31	66.95	66.91
pyrimidine	61.23	61.18	60.88	60.98
pyrrole	57.16	57.27	56.95	57.59
phosphole	77.81	78.18	77.75	79.51
thiazole	61.48	61.91	61.48	62.05
thiophene	67.32	67.50	67.15	67.58
**ME**	−0.01	0.10	−0.25	
**MAE**	0.53	0.45	0.53	
**SD**	0.77	0.73	0.69	

Table S12: Dynamic polarizabilities (a.u.) compared to the CC3 reference. Mean error (ME), mean absolute error (MAE), and standard deviation (SD) of the errors for range-separated methods with tuned RS parameter μ_{opt} in the aug-cc-pVTZ basis set.

	TD-HF-srPBE	TD-CAS-srPBE	ERPA-CAS-srPBE	CC3
$\omega = \mathbf{0.072003}$ a.u.				
benzene	70.66	70.82	70.44	70.86
benzonitrile	90.74	90.90	90.37	88.94
furan	49.89	49.99	49.71	49.75
imidazole	49.99	50.14	49.86	50.65
oxazole	44.27	44.36	44.11	44.32
phenol	77.39	77.56	77.14	76.82
pyrazine	60.99	60.82	60.52	60.89
pyridazine	60.71	60.67	60.37	60.62
pyridine	65.70	65.67	65.32	65.31
pyrimidine	59.85	59.80	59.51	59.62
pyrrole	55.83	55.94	55.63	56.25
phosphole	75.32	75.68	75.30	76.85
thiazole	60.12	60.51	60.13	60.69
thiophene	65.70	65.88	65.55	65.98
$\omega = \mathbf{0.093215}$ a.u.				
benzene	72.49	72.65	72.25	72.63
benzonitrile	93.53	93.73	93.12	91.45
furan	50.98	51.08	50.79	50.80
imidazole	51.05	51.21	50.91	51.76
oxazole	45.13	45.23	44.97	45.16
phenol	79.51	79.69	79.25	78.85
pyrazine	62.62	62.42	62.10	62.50
pyridazine	62.20	62.18	61.86	62.07
pyridine	67.34	67.31	66.95	66.91
pyrimidine	61.23	61.18	60.88	60.98
pyrrole	57.16	57.27	56.95	57.59
phosphole	77.81	78.18	77.75	79.51
thiazole	61.48	61.91	61.48	62.05
thiophene	67.32	67.50	67.15	67.58
**ME**	−0.01	0.10	−0.25	
**MAE**	0.53	0.45	0.53	
**SD**	0.77	0.73	0.69	

References

(S1) Alms, G. R.; Burnham, A.; Flygare, W. H. Measurement of the dispersion in polarizability anisotropies. *J. Chem. Phys.* **1975**, *63*, 3321–3326.

(S2) Kamada, K.; Ueda, M.; Nagao, H.; Tawa, K.; Sugino, T.; Shmizu, Y.; Ohta, K. Molecular Design for Organic Nonlinear Optics: Polarizability and Hyperpolarizabilities of Furan Homologues Investigated by Ab Initio Molecular Orbital Method. *J. Phys. Chem. A* **2000**, *104*, 4723–4734.

(S3) Soscun, H.; Bermudez, Y.; Castellano, O.; Hernandez, J. Effects of protonation on the dipole polarizability of monocyclic azines: a theoretical study. *Chem. Phys. Lett.* **2004**, *396*, 117–121.

(S4) Hinchliffe, A.; Soscún M., H. J. Ab initio studies of the dipole polarizabilities of conjugated molecules: Part 5. The five-membered heterocyclics C_4H_4E (E = BH, AlH, CH_2, SiH_2, NH, PH, O and S). *J. Mol. Struct. (Theochem)* **1995**, *331*, 109–125.
